# Assessment of *BMP7*, *SMAD4*, and *CDH1* Expression Profile and Regulatory miRNA-542-3p in Eutopic and Ectopic Endometrium of Women with Endometriosis

**DOI:** 10.3390/ijms24076637

**Published:** 2023-04-02

**Authors:** Anna Zubrzycka, Monika Migdalska-Sęk, Sławomir Jędrzejczyk, Ewa Brzeziańska-Lasota

**Affiliations:** 1 Department of Biomedicine and Genetics, Medical University of Lodz, St. Pomorska 251, C-5, 92-213 Lodz, Poland; ewa.brzezianska@umed.lodz.pl; 2 Institute of Medical Expertises, St. Aleksandrowska 67/93, 91-205 Lodz, Poland; 3 Operative and Conservative Gynecology Ward, Dr. K. Jonscher Municipal Medical Centre, St. Milionowa 14, 93-113 Lodz, Poland

**Keywords:** endometriosis, expression genes, *BMP7*, *SMAD4*, *CDH1*, miRNA-542-3p, EMT

## Abstract

Alterations in the expression of numerous genes and the miRNAs that are recognized as their regulators in the endometrial cells of women with endometriosis may disrupt the intracellular signaling pathways associated with epithelial–mesenchymal transition (EMT). So far, the functional role of *BMP7* in endometrial physiology has been confirmed, especially in the context of fertility, but the role of the activation of a specific mechanism operating through the BMP–SMAD–CDH1 axis in the formation of endometrial lesions remains unexplored. The aim of this study was to evaluate the expression profile of miR-542-3p and the EMT markers (*BMP7*, *SMAD4*, *CDH1*) in matched eutopic endometrium (EUE) and ectopic endometrium (ECE) samples from women with endometriosis in relation to healthy women. The levels of expression of the studied genes and miRNA in peripheral blood mononuclear cells (PBMCs) obtained from women diagnosed with endometriosis and those without the disease were also evaluated. Fifty-four patients (n = 54: with endometriosis—n = 29 and without endometriosis—n = 25) were included in the study. A comparative analysis of the relative mean expression values (RQ) of the studied mRNA and miRNA assessed by RT-qPCR demonstrated downregulation of *BMP7*, *SMAD4*, and *CDH1* expression in ectopic lesions and upregulation in the eutopic endometrium compared with the control group. In the eutopic tissue of women with endometriosis, miR-542-3p expression was similar to that of the control but significantly lower than in endometrial lesions. We also confirmed a trend towards a negative correlation between miR-542-3p and *BMP7* in ectopic tissue, and in PBMC, a significant negative correlation of miR-542-3p with further BMP signaling genes, i.e., *SMAD4* and *CDH1*, was observed. These results indicate that the miRNA selected by us may be a potential negative regulator of BMP7-SMAD4-CDH1 signaling associated with EMT. The different patterns of *BMP7*, *SMAD4*, and *CDH1* gene expression in ECE, EUE, and the control endometrium observed by us suggests the loss of the endometrial epithelium phenotype in women with endometriosis and demonstrates their involvement in the pathogenesis and pathomechanism of this disease.

## 1. Introduction

Endometriosis is one of the most common gynecological diseases, affecting 5–10% of women of reproductive age [1]. It is characterized by the presence of endometrial-like tissue outside the uterine cavity, which is manifested by chronic pelvic pain and/or infertility in 35–50% of women [2,3,4]. The course of this disease is heterogeneous, with a complex etiopathogenesis involving many genetic, epigenetic, immunological, hormonal, and environmental factors [3,5,6].

Although endometriosis is generally regarded as a benign condition, endometriotic cells demonstrate invasive features, as indicated by their progressive growth, high recurrence rate, and tendency to metastasize [7,8,9]. These abilities are believed to be a prerequisite for the formation of endometriotic lesions. It is suggested that this process is associated, among other factors, with metabolic changes in endometrial cells, the occurrence of new DNA mutations, the heterogeneity of gland-forming cells, the epithelial–mesenchymal transition (EMT), as well as with any combination of the aforementioned factors [10,11].

As demonstrated by earlier studies, EMT is an important mechanism associated with the embryonic development of the body, the dysregulation of which can lead to pathological conditions such as endometriosis, adenomyosis, and carcinogenesis [12]. In the case of endometriosis, this process is believed to play a significant role in its initiation [10,13,14,15,16]. The involvement of numerous proteins, several transcription factors, and EMT-specific signaling pathways in EMT has been suggested [10,15,17,18]. The molecular abnormalities occurring during EMT are accompanied by morphological changes that result in a change from the epithelial phenotype to the mesenchymal phenotype [16,18,19]. In the process, the epithelial cells lose the characteristics that enable differentiation, including intercellular adhesion, apical-basal polarization, and motility dysfunction, acquiring mesenchymal cell properties such as migration, invasion, and resistance to apoptosis [18,19,20]. These are the factors that favor the implantation of endometrial tissue into the abdominal cavity [21].

During EMT, there is a loss of expression of a number of epithelial surface markers, including E-cadherin (*CDH1*), Cytokeratins, Desmoplakin, Mucin-1, and Occludin, while mesenchymal markers, such as N-cadherin, Vimentin, Vitronectin, and Fibronectin are elevated [20,22,23]. E-cadherin is a transmembrane glycoprotein that is responsible for the cell–cell and cell–matrix contacts through adjacent connections. Thus, impairing its expression can lead to increased cell motility, thereby promoting the EMT process and accelerating cellular invasion and tumor progression [20,24,25]. In women with endometriosis, the involvement of E-cadherin in the strong adhesion of endometriotic cells at ectopic sites has been demonstrated [26]. Studies on the expression of E-cadherin in endometriosis cases reported to date have led to inconclusive results. Most studies have reported significantly reduced expression in endometriosis cases compared to the normal endometrium [27,28,29,30,31,32]. However, there are reports that exclude a decrease in the level of E-cadherin in endometriosis [33,34], and even describe its increase in black lesions of the peritoneum and deep pelvic endometriosis [21]. Therefore, it seems necessary to investigate the expression profile of the recognized EMT marker, E-cadherin, in the endometrium and in endometriosis cases.

The ability of endometrial cells to survive in a new location is known to be a prerequisite for the development of endometrial lesions [3]. One of the factors contributing to the survival of ectopic endometrial cells outside their physiological location may be disorders occurring in women with endometriosis, which are associated with the release of bone morphogenetic proteins (BMPs) and their receptors (BMPRs) into the peritoneal fluid [35]. These changes, especially at the early stages of endometriosis, may indicate increased formation of new blood vessels during this period and at subsequent stages of the disease promote the process of angiogenesis, which is considered one of the key stages in the development of endometriosis [3,35]. Increased expression of the gene encoding the BMP7 molecule has been shown in women with heavy menstrual bleeding [36]. This molecule may promote the formation of endometrial implants, probably due to the effect of BMP7 on the intensity of bleeding [37]. Decreased expression of the BMP2-encoding gene in endometrial cells may adversely affect the process of decidualization in women with endometriosis, leading to problems with fertilization and pregnancy [38]. Additionally, by activating appropriate signaling pathways, both BMP molecules and their receptors can affect the processes of migration, apoptosis, and adhesion of vascular endothelial cells and facilitate the growth of endometrial cells, invading even distant parts of the body [35]. 

BMPs, after binding to receptors, activate the transcription factors SMAD1 and/or SMAD5, which then bind to SMAD4 and together translocate to the nucleus, where they control the expression of the target genes in a context-dependent manner [39]. By mediating intracellular signals, SMAD4 activates TGF-β1-stimulated cell migration. In patients with endometriosis, *TGF-β1* and *SMAD4* are upregulated, which may suggest the activation of the TGF-β/SMAD signaling pathway. These results also indicate the possibility of abnormal metaplasia and differentiation, as well as lymphatic spread to form ectopic endometrial tissue via SMAD [40]. In contrast, the reduced expression of *SMAD4* and *BMP-6* in women with peritoneal endometriosis has been associated with impaired granular cell function (ovarian cumulus) [41]. This further supports the idea that both TGF-β and the BMP signaling via the central SMAD4 mediator are key pathways in stromal cells for decidualization, and implantation is provided by studies conducted in a mouse model [42]. 

In patients with endometriosis, diagnosis without histological examination often exposes patients to ineffective treatment. On the other hand, histological abnormalities and the extent of their occurrence may vary among patients. Therefore, molecular analysis and typing of potential specific markers of disease development could support diagnosis. Few studies have assessed the expression of *SMAD4* and *BMP7* in endometriosis. BMP7/SMAD signaling seems to be particularly important in this context due to its antiprofibrotic role through the negative regulation of the TGF-β/SMAD signaling pathway. In turn, the antagonistic effect of BMP7 fibrogenesis can be abolished by decreasing its expression mediated by miR-542-3p, a recognized regulator of the development of liver fibrosis [43], a process that also accompanies endometriosis. Its role in EMT regulation through the direct targeting of *BMP7* in renal fibrosis is also known [44]. It has been suggested that miR-542-3p may be associated with endometrial decidualization [45], and its upregulation has been observed in early endometriosis [46]. Recent studies have shown that miR-542-3p, a tumor suppressor, can inhibit the proliferation and differentiation of tumor cells by downregulating the expression of the survivin protein [47] as well as reducing their invasion by targeting the AKT and BMP signaling pathways [48,49].

Based on the above-documented scientific results, we focused on validating the hypothesis that *BMP7* acts via a direct target of miR-542-3p, and their mutual interaction is involved in the development of endometriosis. At the beginning of our study, based on the 3′-UTR complementary prediction with the Target Scan Human 7.2. database (http://www.targetscan.org/vert_72/ accessed on 1 March 2023), we established that many genes are potential targets of miR-542-3p. Of course, the regulation of gene expression by miRNA occurs through a complex system in which a single miRNA may modulate many transcripts. However, due to the similarity of the miR-542-3p sequence to the 7mer-A1 region in the 3′UTR region of BMP7, the above-mentioned databases suggest a regulatory function of miR-542-3p, especially in the case of modulating *BMP7* expression. More importantly, disturbances in intracellular signaling pathways in endometriosis are strongly associated with the expression modulation of EMT epithelial markers as well as with disturbances in BMPs molecules involving in decidualization in women with endometriosis [35]. Therefore, the aim of this study was to analyze the expression, localization, and correlations of *BMP7*, *SMAD4*, *CDH1*, and miR-542-3p in ectopic and eutopic endometrial tissues from the proliferative and secretory phase endometrium of women with endometriosis as well as in peripheral blood mononuclear cells (PBMCs) obtained from patients with endometriosis and without the disease. It should be pointed out that, currently, the simultaneous expression of miR-542-3p, *BMP7*, *SMAD4*, and *CDH1* has never been evaluated in the same sample or in paired eutopic and ectopic tissues from the same patient. To investigate whether these potentially dysregulated molecules could also be associated with specific clinical features of the disease, we analyzed their expression levels in ectopic lesions from patients with different clinical features and biochemical parameters.

## 2. Results

### 2.1. The Expression Profile of the Studied Genes, BMP7, SMAD4, CDH1, and miR-542-3p, in Ectopic Lesions (ECE) and Eutopic Endometrium (EUE) vs. Control Endometrium (C1)

*BMP7, SMAD4, CDH1,* and miR-542-3p mRNA were expressed in all evaluated tissues. Expression levels in ectopic lesions and matched eutopic endometrium obtained from the same patient were compared to those of controls. In ECE samples, the expression of the studied genes was downregulated. *BMP7* showed a 4.4-fold, *SMAD4* a 2.3-fold, and *CDH1* a 2.9-fold change in expression. However, in EUE samples, the expression levels of all genes were upregulated, with a 2.7-fold change for *BMP7*, a 1.3-fold change for *SMAD4* and a 3.1-fold change for *CDH1*. MiR-542-3p was upregulated in ECE samples with a 2.7-fold change and downregulated in EUE with a 1.1-fold change in expression.

Statistically significant differences in the level of gene expression between the study groups for *BMP7* (*p* = 0.00001), *SMAD4* (*p* = 0.0003) and *CDH1* (*p* = 0.00001), and miR-542-3p (*p* = 0.0018) were found. Significant increases in expression in the EUE samples compared to the ECE samples for *BMP7* (*p* = 0.033528), *SMAD4* (*p* = 0.010696), and *CDH1* (*p* = 0.005565) were confirmed. For *CDH1*, a statistically significant difference in the expression levels between EUE and C1 was also shown (*p* = 0.017259). For the *SMAD4* gene, a negligible trend towards lower expression in ECE than in C1 samples (*p* = 0.064847) was observed. In contrast, the level of expression of miR-542-3p was statistically significantly higher in ECE than in EUE samples (*p* = 0.002233) and in ECE compared to C1 samples (*p* = 0.001480).

**Figure 1:**
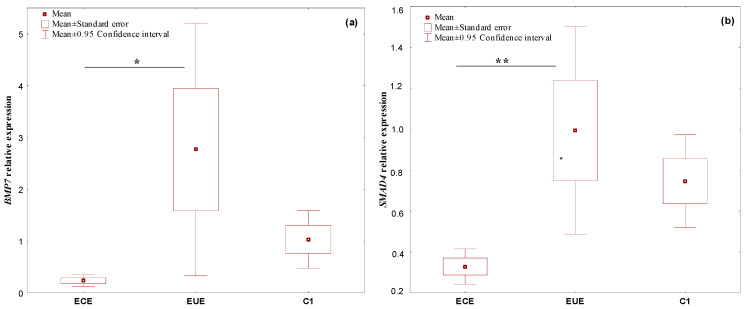

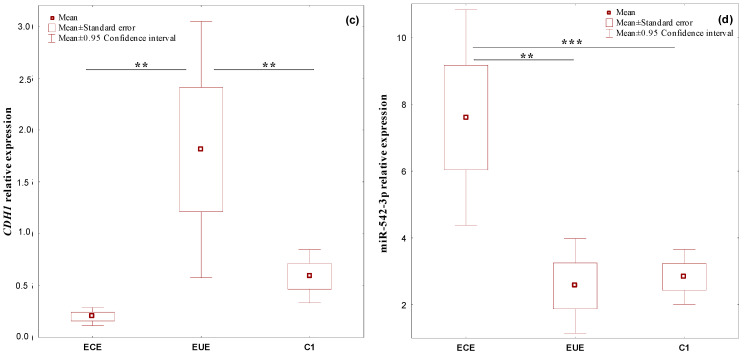
The expression levels (mean RQ values) of the genes (**a**) *BMP7*, (**b**) *SMAD4*, (**c**) *CDH1,* and (**d**) miRNA-542-3p in individual tissue materials (sources of tissue: ectopic endometrium—ECE and eutopic endometrium—EUE, obtained from the same patient with endometriosis) and control endometrium (C1). * *p* < 0.05, ** *p* < 0.01, *** *p* < 0.001; Neuman–Keuls’ multiple comparison test.

The expression levels (mean RQ values) of the studied genes and miRNA in the individual tissue materials (ectopic endometrium—ECE and eutopic endometrium—EUE, obtained from the same patent with endometriosis) and control endometrium (C1) are presented in Figure 1.

### 2.2. Expression Profile of the Studied Genes, BMP7, SMAD4, CDH1, and miR-542-3p, in PBMCs: From Patients with Endometriosis vs. Patients without Endometriosis (C2)

*BMP7*, *SMAD4*, *CDH1,* and miR-542-3p expression levels were assessed in PBMCs from endometriosis patients and in PBMCs from patients without endometriosis, who constituted the control group (C2). *SMAD4, CDH1,* and miR-542-3p mRNA were expressed in all evaluated samples. qPCR results (RQ values) were not obtained for the *BMP7* gene. No statistically significant differences were observed in the level of expression of the studied genes and miR-542-3p in PBMCs between the study groups. The results are shown in Table 1.

### 2.3. Correlations between the Expression Level of the Studied Genes, BMP7, SMAD4, CDH1 and miR-542-3p, in 3 Different Biological Materials (Ectopic Endometrium—ECE, Eutopic Endometrium—EUE and PBMCs) Obtained from the Same Patient with Endometriosis

**Figure 2:**
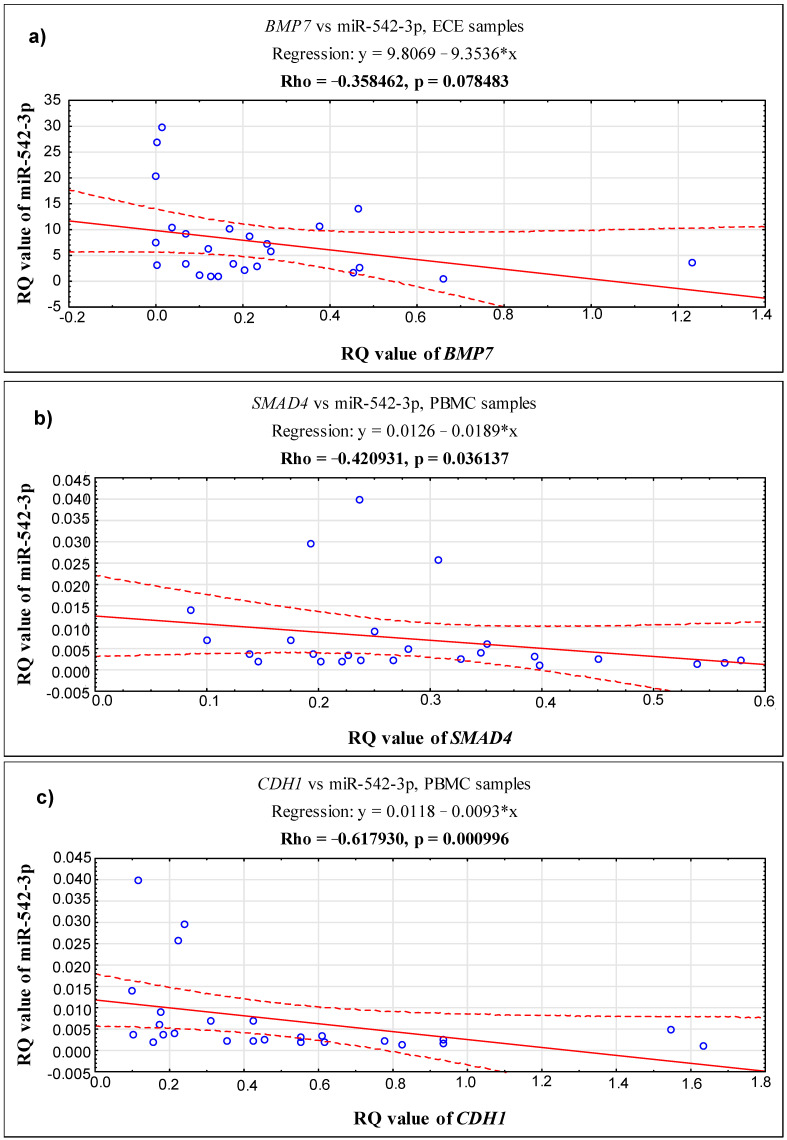
Scatter plots showing correlations between the expression levels (RQ values) of miR-542-3p and genes: (**a**) *BMP7* in ECE samples and (**b**) *SMAD4* and (**c**) *CDH1* in PBMC samples. *p* < 0.05; Spearman’s rank correlation.

In endometrial lesions, a positive correlation was demonstrated between the *BMP7* and *SMAD4* genes (Rho = 0.396154, *p* = 0.049947) and between *BMP7* and *CDH1* (Rho = 0.720769, *p* = 0.000048). In addition, a positive correlation was confirmed between *SMAD4* and *CDH1* (Rho = 0.458462, *p* = 0.021170). For details, see Supplementary Figure S1. The results of the correlation analysis between miR-542-3p and the genes under study indicate a slight trend towards a negative correlation between miR-542-3p and *BMP7* in ectopic tissue (Rho = −0.358462, *p* = 0.078483) (see Figure 2).

A positive correlation was demonstrated between the *SMAD4* and *CDH1* genes in biopsy material derived from the eutopic endometrium (Rho = 0.681538, *p* = 0.000176) and in PBMC patients with endometriosis (Rho = 0.526154, *p* = 0.006900) (see Figure S1). In addition, in PBMC patients, miR-542-3p was negatively correlated with *CDH1* (Rho = −0.617930, *p* = 0.000996) and with *SMAD4* (Rho = −0.420931, *p* = 0.036137) (see Figure 2).

### 2.4. Correlations between the Expression Levels of the Studied Genes, BMP7, SMAD4, CDH1, and miR-542-3p, in Relation to the Clinical Characteristics and Biochemical Parameters of Patients with Endometriosis

The RQ values obtained for the *BMP7*, *SMAD4*, and *CDH1* genes and miR-542-3p in ectopic tissue were analyzed in relation to the following clinical features of patients with endometriosis: age at time of diagnosis, stage of endometriosis (according to rASRM classifications), pelvic pain symptoms (according to the numerical rating scale—NRS), and concentrations of the biochemical parameters of CA-125 and HE4. The results are shown in Table 2.

The analysis of the expression levels of the tested genes and miR-542-3p in the group of patients with endometriosis according to age showed an insignificant upward trend in the expression levels of all tested genes in patients aged ≤40 years compared to older patients. In the case of miR-542-3p, the level of expression was similar in both age groups.

The analysis of the expression levels of *BMP7*, *SMAD4*, *CDH1*, and miR-542-3p according to the stage of endometriosis demonstrated a statistically significant increase in the level of *BMP7* expression in the group of patients with stage III compared to stage IV endometriosis (*p* = 0.035602). Similarly, the level of expression of *SMAD4* was greater in patients with a lower stage (III vs. IV) of the disease, but this result did not reach statistical significance. Positive correlations were observed between the levels of expression of *CDH1* and miR-542-3p and the stage of the disease, but no statistically significant differences were shown between the study groups.

The analysis of the expression levels of the tested genes and miR-542-3p according to pain severity showed an insignificant downward trend in the expression levels of *CDH1* and miR-542-3p in patients with very severe pain symptoms of endometriosis. In contrast, the levels of expression of the *BMP7* and *SMAD4* genes were positively correlated with the degree of pain severity, but for the *BMP7* gene, a statistically significant increase in expression in the group of patients with very severe pain symptoms was confirmed (*p* = 0.017147), while for *SMAD4*, only a negligible upward trend in the expression was observed in this group of patients (*p* = 0.098865).

Division of the group of patients with endometriosis according to the concentration of CA-125 into ≤65 U/mL and >65 U/mL subgroups confirmed a statistically insignificant increase in the levels of expression of *BMP7* and *SMAD4* in the group of patients with a higher concentration of CA-125, while the expression levels of *CDH1* and miR-542-3p were higher in the group of patients with CA-125 ≤ 65 U/mL. In contrast, statistically insignificant increases in gene and miRNA expression were correlated with higher HE4 concentrations in the patients.

### 2.5. Correlations between the Expression Levels of the Studied Genes, BMP7, SMAD4, CDH1, and miR-542-3p, in Relation to the Phase of the Menstrual Cycle in Ectopic Lesion (ECE), Eutopic Endometrium (EUE), and Control Endometrium (C1) Samples

The RQ values obtained for the *BMP7*, *SMAD4*, and *CDH1* genes and miR-542-3p in ECE, EUE, and C1 tissues were analyzed in relation to the phase of the menstrual cycle. The results are shown in Table 3.

In the ectopic endometrium (ECE) samples collected from patients with endometriosis in the proliferative phase of the menstrual cycle, insignificant upward trends in the expression levels of *SMAD4* and miR-542-3p and insignificant downward trends in the expression levels of *BMP7* and *CDH1* were observed compared to tissue samples from patients in the secretory phase. There were also no statistically significant differences in the level of expression levels of the studied genes and miR-542-3p between the proliferative, early, middle, and late secretory phases of the menstrual cycle.

In paired eutopic endometrium (EUE) samples collected from patients with endometriosis in the proliferative phase of the cycle, insignificant upward trends in the expression levels of the studied genes and miR-542-3p compared to tissue samples from patients in the secretory phase was observed. There were also no statistically significant differences in the levels of expression of the *BMP7*, *SMAD4*, and *CDH1* genes and miR-542-3p between the proliferative, early, middle, and late secretory phases of the menstrual cycle.

In control endometrium (C1) samples collected from women in the proliferative phase of the cycle, an insignificant downward trend in the expression of *SMAD4* was observed compared to tissue samples from women in the secretory phase. In the case of *CDH1* and miR-542-3p, the expression levels were similar in the proliferative phase and secretory phase of the cycle. In the case of *BMP7* a statistically significant increase in the level of expression in the group of women in the proliferative phase of the cycle compared to those in the secretory phase was observed (*p* = 0.001909). There were also statistically significant differences in the level of expression of *BMP7* between the proliferative, early, middle, and late secretory phases of the menstrual cycle (*p* = 0.0284). The post-hoc analysis confirmed a statistically significant increase in *BMP7* expression in the proliferative phase of the cycle compared to in the early (*p* = 0.004526), middle (*p* = 0.003339), and late (*p* = 0.003403) secretory phases of the menstrual cycle.

## 3. Discussion

Endometriosis is a disease with a complex etiology, including genetic factors, in which disturbed cell signaling may contribute to changes in the expression of many genes and the miRNAs that regulate them. These changes contribute to the formation, development, and spread of endometrial foci. There is a growing body of evidence linking the pathogenesis of endometriosis to a polygenic disorder, but none of the genes have been validated as markers for use in the diagnosis of endometriosis [50]. Many miRNAs have also been studied as potential biomarkers of endometriosis [51,52,53,54,55,56]. However, their significance in this disease and participation in possible malignant transformation remain unexplored [57,58]. Research evidence suggests that the development of endometriosis may be facilitated by endometrial dysfunction involving both eutopic (EUE) and ectopic endometrium (ECE), associated with molecular changes within the endometrial cells [59,60]. Therefore, the presented study focusef on determining the expression profile of miR-542-3p and EMT markers (*BMP7*, *SMAD4*, *CDH1*) in both tissues in relation to endometrium samples from healthy women. The expression levels of selected genes and miRNA in peripheral blood mononuclear cells (PBMCs) from women diagnosed with endometriosis were compared to those of controls. Our results suggest that the loss of the endometrial epithelium phenotype is reflected by the differentiated expression of the *BMP7*, *SMAD4*, and *CDH1* genes and miR-542-3p between the ectopic and eutopic endometrium. Additionally, based on the obtained results, we conclude that the BMP7–SMAD4–CDH1 signaling associated with the EMT process may be of key importance in the etiopathogenesis of endometriosis, and miR-542-3p may be a potential negative regulator of the BMP7–SMAD4–CDH1 axis.

Several studies have focused on the importance of BMP/SMAD signaling in endometriosis [41,61,62] as well as on the role of miRNA in the development and progression of the disease [57,58,63,64]. Among the many candidates, one potentially interesting miRNA is miR-542-3p, for which a suppressor function in tumor development is well characterized. Its decreased expression is associated with tumor progression via C-src-associated oncogenic pathways [65,66]. In the endometrium, the role of miR-542-3p is unclear. Only one study has reported that a decrease in its expression in eutopic endometrial stromal cells enables morphological and biological differentiation of the endometrium [45]. In our study, we did not confirm differences in the level of expression of miR-542-3p between the eutopic tissue of women with endometriosis and that of control subjects. Similarly, in PBMC samples, the expression profile of miR-542-3p did not differ between affected women and healthy women; however, we observed an increase in the expression of this miRNA with an increase in the HE4 marker in affected women. It is known from the literature on this topic that the concentration of HE4 is elevated in women with endometriosis, especially after the rupture of chocolate cysts, accompanied by spillage of the contents into the pelvic cavity [67], and the highest values have been observed in ovarian cancer patients [67,68].

To date, several studies have suggested that changes in serum and plasma levels of specific circulating miRNAs, including miR-542-3p, represent a potential diagnostic tool for the detection of endometriosis, especially when two or more miRNAs can be evaluated simultaneously. A decrease in the expression of circulating miR-542-3p was found to be particularly informative as a biomarker of endometriosis in optimal combinations with other miRNAs (e.g., miR-199, miR-122, miR-145). The obtained high sensitivity and specificity values of this marker panel are 93.22% and 96.00%, respectively [69,70]. This result encouraged us to determine the role of miR-542-3p in the context of a noninvasive diagnostic tool using PBMCs along with the evaluation of miRNA expression in a panel with other genes.

An interesting result from our study is the demonstration of a significant increase in miR-542-3p expression in endometrial foci, which is consistent with the findings of other authors [71,72]. This result is important in terms of the ability of ectopic endometrial stromal cells to decidualize. It has been postulated that the overexpression of miR-542-3p inhibits the process of differentiation (decidualization) of endometriotic stromal cells [72,73,74] and the proliferation of human endometrial stromal cells [75]. An important result from our study is the observation of a positive, albeit statistically insignificant, correlation between miR-542-3p expression and the severity of the disease. Thus, our findings and those of other authors point to the possibility of using miR-542-3p as a potential therapeutic target in endometriosis. Since endometriotic cells exhibit biological behavior characterized by increased invasiveness, similar to cancer, in the course of endometriosis [76], it is notable that the elevated expression of miR-542-3p has been associated with the possibility of preventing the development of ovarian cancer. The increased expression of this miRNA has been shown to inhibit the proliferation, migration, and invasion of cancer cells in vitro [77]. The aforementioned results of other authors emphasize the legitimacy of using changes in miR-542-3p expression to understand the molecular mechanisms involved in the development of endometriosis.

The evidence provided by the published studies indicates that miR-542-3p inhibits osteogenic differentiation and promotes osteoblast apoptosis by repressing *BMP7* and its subsequent signaling [48]. Similarly, in cancer cells, miR-542-3p inhibits their invasion by targeting the activation of the AKT pathway and BMP signaling pathways [49]. BMP signaling has been found to be a direct target of miR-542-3p. To the best of our knowledge, the current study is the first for which the results suggest that miR-542-3p may be involved in the development of endometriosis by activating BMPs signaling. After analyzing the results of the relationship between miR-542-3p and the studied genes, we identified a tendency towards a negative correlation between miR-542-3p and *BMP7* in ectopic tissue.

BMP7 is a multifunctional secretory protein with different biological activities. It participates in many cellular processes, such as the regulation of proliferation, differentiation, and apoptosis. It plays a key role in the formation and repair of damaged tissues and organs and in embryonal development [78,79]. In the granulosa cells of the ovarian follicle, BMP7 inhibits apoptosis [79]. Several studies support the functional role of BMP7 in endometrial physiology [80,81,82]. A complex regulatory mechanism has been suggested for the inhibition of decidualization in the endometrium by BMP7 and the role of BMP7 as an antiproliferative factor of endometrial stromal cells [83].

In our work, we focused on assessing the expression of *BMP7* mRNA in the eutopic endometrium of women with endometriosis, confirming its significant upregulation compared to the control group. Other authors also showed increased expression of *BMP7*, but before implantation, during the receptive period, compared with its baseline concentration in endometrial epithelial cells in mice [84]. Additionally, in the human endometrium, the expression of this gene was observed in cultured endometrial stromal cells with a high degree of decidualization [82]. However, in advanced stages after the period of endometrial receptivity, *BMP7* mRNA was generally reduced or lost from the uterine epithelium soon after implantation [80,81,82]. In view of our results, as well as the results presented above, comparing the *BMP7* expression pattern in endometriosis and reproduction, seems to be very difficult. However, it is possible to speculate and present a common mechanism whereby high expression of *BMP7* in EUE in patients with endometriosis may be one of the causes of infertility in these women due to endometrial receptivity disorder. Human chorionic gonadotropin and progesterone, the production of which is inhibited by *BMP7* from trophoblasts, are known to be extremely important for maintaining pregnancy [85]. Thus, the presence of *BMP7* may promote endometrial dysfunction and thus become problematic for attacking trophoblasts. Therefore, reduced expression of *BMP7* may be necessary, not only for the development of the receptive endometrium, but also for trophoblast invasion to sustain pregnancy, which further supports our hypothesis about the role of *BMP7* (its upregulation) in fertility disorders of women with endometriosis.

The dependence of *BMP7* expression in endometrial tissues on progesterone and its cyclicality, with significantly low expression in the middle and late secretory phase and in early pregnancy compared to its expression in the middle phase of proliferation, is interesting [83]. By analyzing the results of our research, we confirmed the above observations by showing a significant difference in *BMP7* expression in the normal endometrium between the proliferative phase, in which the level of expression of the studied gene was the highest, and the secretory phase of the cycle, with the lowest expression occurring in its early phase. In the eutopic tissue of women with endometriosis, or in the endometrial foci, the expression profile of *BMP7* remained unchanged throughout the menstrual cycle, indicating physiological differences in the normal endometrium compared to pathological endometrial tissue.

With regard to the clinical symptoms of endometriosis, we confirmed a significant increase in *BMP7* expression with an increase in pain severity in patients with endometriosis. There is little data on the role of *BMP7* in the development of chronic pelvic pain, and the mechanisms governing the pathophysiology of its development remain unexplored. This process is likely to be associated with the predominance of the inflammatory environment locally in the endometrial tissue in the form of an increase in released proinflammatory cytokines, which results in the stimulation of sensory nerves and altered activation of nociceptive pathways [4]. Moreover, as a result of heavy retrograde menstrual bleeding—one of the clinical symptoms of endometriosis—fibrosis reactions occur, leading to the formation of local scars and adhesions, accompanied by severe pain. It has been indicated that *BMP7* affects the intensity of menstrual bleeding, and prolonged menstruation promotes the implantation of endometrial cells from heavy retrograde bleeding [35,36]. Thus, the disturbed expression profile of *BMP7* may suggest its indirect involvement in the mechanism of pelvic pain in women with endometriosis. In addition, the dysregulation of *BMP7* expression in the endometrium can alter metabolic pathways, consequently leading to fibrosis and increasing the formation of adhesions with the progression of endometriosis. In our study, *BMP7* downregulation was noted in severe vs. moderate endometriosis, which could be related to the fact that this gene is involved in angiogenesis, considered to be one of the pivotal stages in the development of endometriosis [35].

An interesting result from our work is the significant reduction in *BMP7* expression in endometrial lesions (ECE) as opposed to in paired EUE tissues and normal endometrium. This result may suggest a loss of the endometrial epithelium phenotype expressed by the heterogeneous expression of *BMP7* in ectopic and eutopic endometrium samples. In view of the fact that *BMP7* is a multifunctional growth factor belonging to the TGF-β superfamily with anti-inflammatory and antifibrotic properties [86], its involvement in the development of endometriosis may coincide with the EMT and be associated with BMP7 signaling dysregulation that is dependent on the SMAD4 signal loop. The low level of *BMP7* expression in ectopic endometrium shown in our study may suggest that BMP/SMAD signaling has not fulfilled its anti-inflammatory and antifibrotic roles in ECE, which may result in the progression of the already implanted endometrial foci.

In our study, we investigated the expression of *SMAD4* in the endometrium of women with endometriosis. The expression level of this gene was significantly lower in ECE compared to in matched EUE samples of women with endometriosis, and it was lower than in controls, which is consistent with the report by Mabuchi et al. [87]. The authors of other studies also demonstrated reduced expression of the *SMAD4* transcript but in the ovarian cumulus cells from women with peritoneal endometriosis-associated infertility compared with the control group [41], as well as in a rat model of intrauterine adhesion [86]. The convergence of our achievements with previous observations also applies to the expression profile of *SMAD4* in eutopic endometrium (EUE) samples of affected women, in which the over-regulation of this gene has been demonstrated [40]. There are also studies in which the levels of both mRNA and the SMAD4 protein were similar in the group with endometriosis and in controls, but unlike us, the results of that study are restricted to the proliferative phase of the menstrual cycle and are limited by a lack of subgroup analyses or adjustment of results for different endometriotic lesions and disease stages [88]. As shown in the research, *SMAD4* is a well-characterized tumor suppressor that participates in carcinogenesis through numerous mechanisms, such as the induction of cell cycle arrest, apoptosis, angiogenesis, and the EMT [89,90]; however, very few studies have looked into the role and mechanism of action of *SMAD4* in endometriosis. Although we did not investigate these mechanisms, the different patterns of *SMAD4* expression shown for the ECE and EUE samples in comparison with the control samples indicates that endometrial cell function may be altered in women with endometriosis. Additionally, since *SMAD4* is a central and critical component of both TGF-β and BMP signaling [89], based on our observations, it can be hypothesized that if its expression is disturbed in endometriosis, the functionality of all proteins of this pathway may be changed.

*SMAD4* has also been recognized as the central component of EMT, which binds to transcription factors (e.g., TWIST1, SNAIL, SLUG) to reduce E-cadherin (CDH1) and alter the epithelial phenotype expression [91]. In recent years, evidence that endometriosis may be associated with impaired expression of the genes important to the EMT process has emerged [10,15,17]. In view of the fact that the characteristic feature of EMT is the functional loss of E-cadherin expression in epithelial cells [18,22] and, in turn, that EMT is the prerequisite for the primary establishment of endometrial lesions [14], we evaluated the expression profile of this gene in ectopic and eutopic endometrium samples of women with endometriosis and in control samples. As shown in comparative studies [33,34], the expression profile of *CDH1* did not change in the endometrial foci and control endometrium. However, we found that *CDH1* expression was significantly reduced in ectopic lesions compared to in the eutopic endometrium of patients in paired samples, which is consistent with the reports of other authors [27,30]. Decreased expression has been observed not only in ovarian endometriosis, as in the present study, but also in peritoneal endometriosis, whereby the number of E-cadherin-negative epithelial cells was greater than in the eutopic endometrium [76,92]. In in vitro models, endometriotic cells are deficient in E-cadherin, which allows their detachment from the original site as well as increased migration and invasion [28,76,92]. The loss of E-cadherin expression is known to lead to both the breakdown of the E-cadherin–catenin complex and the growth of free cytoplasmic β-catenin, which may trigger the expression of EMT-inducing transcription factors, consequently allowing endometrial cells to adhere to pelvic implantation sites [93]. Therefore, based on our achievements, in combination with those of others, we speculate that the loss of E-cadherin expression, leading to mesenchymal morphology, may be a key mechanism in the pathogenesis of endometriosis.

A deeper understanding is required regarding the potential use of *CDH1* as a diagnostic marker of endometriosis. A few studies have reported significantly increased expression of *CDH1* in the normal uterine endometrium in the secretory phase compared to in ovarian endometriosis [94]. In our experiment, the expression pattern of *CDH1* did not differ depending on the phase of the menstrual cycle in either ectopic tissue or the control endometrium. The controversial nature of the results can be explained by the heterogeneity of the cellular material used in the study and the size of the analyzed groups of patients in the individual phases of the menstrual cycle. Some authors have reported a negative correlation between *CDH1* expression and the presence of deep infiltrative endometriosis [31]. We observed a negative correlation of *CDH1* expression levels with pelvic pain symptoms and a positive correlation with the progression of endometriosis, although without statistical significance for either clinical feature of the disease. Taking into consideration the little progress made in early diagnosis or the prediction of the progression of endometriosis, we propose a focus in further studies on determining the correlation of the *CDH1* expression pattern with the clinical and pathological outcomes and using these results to improve clinical management.

In summary, despite significant advances in the treatment of endometriosis, biomarkers of this disease are still being sought, but studies in this area are burdened by methodological difficulties. The exact course of the pathogenetic cascade in endometriosis still remains largely unknown. Nevertheless, it is generally accepted that disturbances in the expression profiles of genes and regulatory miRNAs with the consequence of the inhibition or activation of signal transduction pathways in endometrial stromal cells are the key factors associated with the formation and spread of endometrial foci. In the present study, a comparative analysis of the relative mean expression values of *BMP7*, *SMAD4*, *CDH1*, and miR-542-3p in patient samples from the ectopic endometrium, its eutopic counterpart, and from endometrium samples of healthy women demonstrated a downregulation in the expression levels of the studied genes in ectopic lesions and an upregulation in the eutopic endometrium compared with controls. In women with endometriosis in the eutopic tissue, the expression of miR-542-3p was close to that of the control but significantly lower than in endometrial lesions. We also confirmed a negative correlation trend between miR-542-3p and *BMP7* in ectopic tissue, and in PBMCs, a significant negative correlation of miR-542-3p with further BMP signaling genes, i.e., *SMAD4* and *CDH1*, was identified. This indicates that the miRNA selected by us may be a potential negative regulator of the BMP7–SMAD4–CDH1 axis. Differentiated expression of the *BMP7*, *SMAD4*, and *CDH1* genes—the components of the aforementioned signaling between paired ectopic and eutopic endometrium in women with endometriosis—may indicate their involvement in the pathogenesis and pathomechanism of endometriosis. However, further studies need to be conducted to elucidate the exact cause of the imbalance in the expression levels of the genes and miRNA studied and to investigate the regulatory mechanism of the BMP7–SMAD4–CDH1 signaling pathway associated with miR-542-3p in endometriosis.

As for the strengths of our work, we have emphasized the importance of the miR-542-3p–BMP7–SMAD4–CDH1 axis in the pathogenesis of endometriosis. A novelty of our work was the analysis of miR-542-3p, *BMP7*, *SMAD4*, and *CDH1* expression in PBMCs obtained from women diagnosed with endometriosis compared to those obtained from healthy women. Contrary to our expectations, we observed no differences in the expression profile of the studied genes and miRNA in PBMCs. The results should not be entirely surprising, because in the case of genes involved in inflammatory processes and the autoimmune response, it has been demonstrated that the gene profile of leukocytes in women with gynecologic disease is similar to that of other nongynecologic and chronic inflammatory diseases [95], powerfully supporting the acute impact of endometriosis on the systemic immunological status. The lack of clear differences in the gene expression in PBMCs from women with and without endometriosis may suggest that endometriosis should be perceived primarily as a local disease for which only some molecular changes are reflected in gene expression at the systemic level.

A limitation of our project is the fact that only small subgroups of patients were included in the study. This may also be the reason why relatively few statistically significant results have been obtained with respect to the associations between *BMP7*, *SMAD4*, *CDH1*, and miR-542-3p expression levels and the biochemical parameters and clinical features of endometriosis. In addition, at the present stage of research, we have not been able to conduct experiments verifying the mechanism of action of miR-542-3p in the BMP7–SMAD4–CDH1 signaling pathway and its relationship with the EMT process. Therefore, in vitro and in vivo analyses are needed to investigate the causality of our results and to understand the in-depth mechanisms of the interactions of miR-542-3p with the genes of the extensive BMP7–SMAD4–CDH1 signaling network.

## 4. Materials and Methods

### 4.1. Research Ethics

The protocols of this study were approved by the Bioethics Committee of the Medical University in Lodz (resolution No. RNN/67/19/KE, 12 February 2019). All participants were fully informed and signed individual consent forms prior to participation in the study. All methods were implemented in accordance with the relevant guidelines and regulations. The study was conducted in accordance with Good Clinical Practice and the principles of the Helsinki Declaration.

### 4.2. Clinical Groups

The study cohort included 29 women with endometriosis (mean age: 38.38, SD: 6.83 years; age range: 22–49 years) and 25 (mean age: 44.68, SD: 3.34 years; age range: 38–44 years) without endometriosis (control group). During the elective diagnostic or therapeutic laparoscopy for suspected extrauterine endometriosis due to pain symptoms and/or infertility investigation, biological material was obtained from the patients at the Operative and Conservative Gynecology Ward, Dr. K. Jonscher Municipal Medical Centre, Lodz, Poland, who were admitted in the period of March 2019–May 2020. For all patients the inclusion criteria was a diagnosis of endometriosis with simultaneous surgical treatment. The exclusion criteria for all participants were the presence of adenomyosis; cancer of the uterus or cervix; other diseases of the uterus, fallopian tubes, and ovaries; and pregnancy, breastfeeding, or hormone treatment for at least 12 months prior to the time of surgery. According to the criteria laid down by the revised American Fertility Society guidelines [96], regardingthe stage of endometriosis among the examined patients (n = 29), 4 groups were histopathologically confirmed: I (n = 1) and II (n = 2), III (n = 16) and IV (n = 10). Additionally, women with endometriosis reported mild (n = 11), moderate (n = 2), or severe (n = 16) pelvic pain, which was distinguished using the numerical rating scale (NRS). Before surgery, the fertility status and concentration data for cancer antigen (CA)-125 and human epididymis protein 4 (HE4) were collected. CA-125 results were not available for 3 patients, and HE4 results were not available for 14 patients. 

Control samples were collected from patients with uterine myomas who underwent a hysteroscopy or laparoscopic myomectomy without visual evidence of endometriosis and adenomyosis. During the surgery, the absence of endometriosis and adenomyosis was confirmed by meticulous examination of the pelvic and extra-pelvic peritoneum, ovaries, intestine, and diaphragm. Histopathological evaluations confirmed a normal endometrium in all controls. The patients in the control group did not suffer from endometriosis. 

### 4.3. Sample Collection and RNA Isolation

The following biological material was obtained from the same patient with endometriosis from 3 locations (paired samples): (1) a piece of tissue was taken from an ovarian endometrial cyst (n = 27) or sections from the peritoneum of the small pelvis (n = 2) for the ectopic endometrium (ECE) samples; (2) a piece of the mucosa/endometrium from the uterine cavity was obtained by biopsy (n = 25) for the eutopic endometrium (EUE) samples (no biopsies were obtained from 4 patients with endometriosis); and (3) whole blood from which peripheral blood mononuclear cells (PBMCs) were isolated (n = 25) (whole blood was not obtained from 4 patients with endometriosis). Paired EUE and ECE tissues and PBMCs were obtained from women who presented with mild to severe endometriosis (n = 25). The phases of the menstrual cycle for these patients were determined on the basis of the histological evaluation of the endometrium and the date of the patient’s last menstruation. Seven (7) patients were in the proliferative phase, and eighteen (18) were in the secretory phase of their cycle, which was divided into early (n = 7), middle (n = 4), and late (n = 7).

Among the women in the control group, six (6) patients were in the proliferative phase, and nineteen (19) were in the secretory phase of the cycle, which was divided into early (n = 5), middle (n = 5), and late (n = 9). Paired samples were obtained from each woman, including: (1) a piece of the normal endometrial tissue from the uterine cavity obtained by biopsy (n = 25; C1) and (2) whole blood, from which PBMCs were isolated (n = 25; C2). 

The biological material was secured and prepared according to the protocol described in the previous article [97]. Isolation of total RNA from eutopic, ectopic, normal endometrial tissue, and peripheral blood mononuclear cells (PBMCs) was performed with the same types of isolation kits as before. Qualitative and quantitative evaluations of the isolated RNA were performed by spectrophotometry (260/280 nm), using an Eppendorf BioPhotometerTM Plus apparatus (Eppendorf, Hamburg, Germany).

### 4.4. Relative Gene and miRNA Expression (RQ)

The reverse transcription (RT) reaction for genes was performed using the High-Capacity cDNA Reverse Transcription Kit (Applied Biosystems, Carlsbad, CA, USA) in a Personal Thermocycler (Eppendorf, Hamburg, Germany). The relative gene expression was assessed by real-time polymerase chain reaction (qPCR) using a 7900HT Fast Real-Time PCR System apparatus (Applied Biosystems, Carlsbad, CA, USA). We applied the TaqMan^®^ Gene Expression Assay for the following genes: *BMP7* (Hs00233476_m1), *SMAD4* (Hs00929647_m1), *CDH1* (Hs01023895_m1) and *GAPDH* (Hs99999905_m1), selected as the reference gene in the qPCR reaction.

miRNA reverse transcription was carried out in a Personal Thermocycler (Eppendorf, Hamburg, Germany) using a TaqMan^®^ MicroRNA Reverse Transcription Kit (Applied Biosystems, Carlsbad, CA, USA) with specific RT primers (small RNA-specific RT primers) included in individual TaqMan^®^ MicroRNA Assays: hsa-miR-542-3p (UGUGACAGAUUGAUAACUGAAA) and RNU6B (CGCAAGGATGACACGCAAATTCGTGAAGCGTTCCATATTTTT) as the endogenous control (Applied Biosystems, Carlsbad, CA, USA). Assays for the miRNA hsa-miR-542-3p and RNU6B were used in the qPCR reaction. The components of the reactions and conditions employed were in accordance with the protocol described in a previous article [97].

The relative expression levels of the analyzed genes/miRNA were evaluated by the delta-delta CT method (TaqMan Relative Quantification Assay software, Applied Biosystems, Carlsbad, CA, USA) and are presented as RQ values relative to the *GAPDH*/RNU6B reference genes/miRNA, respectively. The following formula was used to determine the ΔΔCT value: ΔΔCT = ΔCT test sample − ΔCT calibrator sample. For the calibrator (RNA isolated from biological material from a healthy patient without endometriosis), the RQ (relative quantification) value was considered to be equal to 1. In the case of the test samples, an increase in expression was recognized when the RQ value was more than 1, and decreased expression was defined as an RQ value of less than 1.

### 4.5. Statistical Analysis

The statistical analysis was carried out using the Statistica 13.1 program (StatSoft, Cracow, Poland). The Shapiro–Wilk test showed that data were not normally distributed. In order to look for the statistical significance between the analyzed groups, the Mann–Whitney U-test and/or Kruskal–Wallis test was used, depending on the size of the groups. Neuman–Keuls’ multiple comparison test was used to identify possible significant differences in RQ values between the individual variables. The Spearman rank correlation coefficient was used to measure the direction and strength of the relationship for individual variables and the potential relationships between RQ genes and miRNA. The level of correlation was fixed in the following categories: very strong (Rho ≥ 0.80), moderate (Rho = 0.60–0.79), fair (Rho = 0.30–0.59), and poor (Rho ≤ 0.29) [98] For all statistical analyses, statistical significance was assumed at *p* < 0.05. The RQ values, presented as means, for the studied genes/miRNA, were used to calculate the fold changes in gene/miRNA expression in the case group in relation to the control group.

## 5. Conclusions

A heterogeneous profile of the expression levels of the studied genes (*BMP-7*, *SMAD4* and *CDH1*) and miR-52-3p in the ectopic endometrium with respect to its eutopic counterpart suggests the loss of endometrial epithelium phenotype in women with endometriosis. The observed significantly high level of expression of miR-542-3p with simultaneous downregulation of *BMP7*, *SMAD4*, and *CDH1* mRNA transcripts in the endometrial foci may be suggestive of the suppressive function of this miRNA in BMP7–SMAD4–CDH1 signaling. It should be noted that the roles of *BMP7*, *SMAD4*, and *CDH1* in the endometrium appear to be complex and definitely require further study, but these data, including previous evidence, indicate that post-transcriptional regulation of these genes may play an important role in the development and spread of endometriosis.

## Figures and Tables

**Table 1:** The expression levels (mean RQ values) of the studied genes and miR-542-3p in the PBMCs in the group of patients without endometriosis (C2) vs. the group of patients with endometriosis.

Patients	RQ (Mean) in PBMCs			
	*BMP7*	*SMAD4*	*CDH1*	miR-542-3p
without endometriosis (C2)	below detection level	0.260932	1.040384	0.005000
with endometriosis	below detection level	0.289032	0.507280	0.007132
*p* value, Mann-Whitney U test	-	0.396546	0.969326	0.254776

**Table 2:** Expression levels (mean RQ value) of study genes and miRNA in ectopic tissue in relation to clinical features and biochemical parameters of patients with endometriosis.

Features (n)	RQ (Mean)			
	*BMP7*	*SMAD4*	*CDH1*	miR-542-3p
Age: years				
≤40 (18)	0.37679	0.45984	0.62759	6.94957
>40 (11)	0.27315	0.27777	0.19401	6.96345
*p* value	0.411953	0.203814	0.188094	0.610874
rASRM				
III (16)	0.49299	0.43848	0.34562	6.59889
IV (10)	0.12247	0.38937	0.70835	7.71903
*p* value	0.035602 *	0.165427	0.335952	0.979370
pelvic pain symptoms:				
mild (11)	0.11175	0.35783	0.64395	7.29089
severe (16)	0.49299	0.43848	0.34562	6.59889
*p* value	0.017147 *	0.098865	0.194506	0.942037
CA-125 [U/mL]:				
≤65 (10)	0.232350	0.413860	0.699980	7.789560
>65 (16)	0.424319	0.423175	0.350850	6.554813
*p* value	0.451781	0.451781	0.586471	0.516940
HE4 [pmol/L]				
≤50 (8)	0.207938	0.399375	0.170100	8.74418
>50 (7)	0.510286	0.438443	1.182729	10.72834
*p* value	0.231857	0.535820	0.335664	0.955089

* statistically significant *p* < 0.05; Mann–Whitney U test. Abbreviations: rASRM: Revised American Society for Reproductive Medicine classification system for endometriosis.

**Table 3:** Expression levels (mean RQ values) of the studied genes and miRNA in ectopic, eutopic endometrium, and control tissues in relation to the phase of the menstrual cycle.

Endometrium	Cycle Phase	RQ (Mean)			
		*BMP7*	*SMAD4*	*CDH1*	miR-542-3p
ECE	proliferative (7)	0.204629	0.384071	0.194557	8.038829
	secretory (18):	0.247628	0.307778	0.204900	7.433933
	early (7)	0.317100	0.323729	0.233671	6.082714
	middle (4)	0.345050	0.459325	0.156425	7.506125
	late (7)	0.122486	0.205229	0.203829	8.743900
	*p* value	0.4063	0.2282	0.8275	0.9088
EUE	proliferative (7)	5.585586	1.281043	2.404900	3.252214
	secretory (18):	1.672767	0.882044	1.580817	2.297389
	early (7)	1.198814	0.669300	1.266386	1.769357
	middle (4)	0.890000	0.588050	1.397600	0.699525
	late (7)	2.594014	1.262786	1.999943	3.738486
	*p* value	0.4570	0.8672	0.8823	0.3525
C1	proliferative (6)	2.675633	0.487233	0.575633	2.858267
	secretory (19):	0.513374	0.828247	0.595095	2.825600
	early (5)	0.331440	0.707440	1.032660	3.428560
	middle (5)	0.411400	1.074680	0.461920	1.913120
	late (9)	0.671100	0.758456	0.425989	2.997556
	*p* value	0.0284 *	0.2270	0.4436	0.6826

* statistically significant *p* < 0.05; Kruskal–Wallis test.

## Data Availability

Data will be made available on request.

## Supplementary Materials

The following supporting information can be downloaded at: https://www.mdpi.com/article/10.3390/ijms24076637/s1.
