# Supplementary material for: Assessment of BMP7, SMAD4, and CDH1 Expression Profile and Regulatory miRNA-542-3p in Eutopic and Ectopic Endometrium of Women with Endometriosis
Source: Int J Mol Sci. 2023 Apr 2;24(7):6637. doi: 10.3390/ijms24076637 (PMC10095043; doi:10.3390/ijms24076637)
Supplement: Supplementary file 1 [file ijms-24-06637-s001.zip › ijms-2292566-supplementary.pdf]

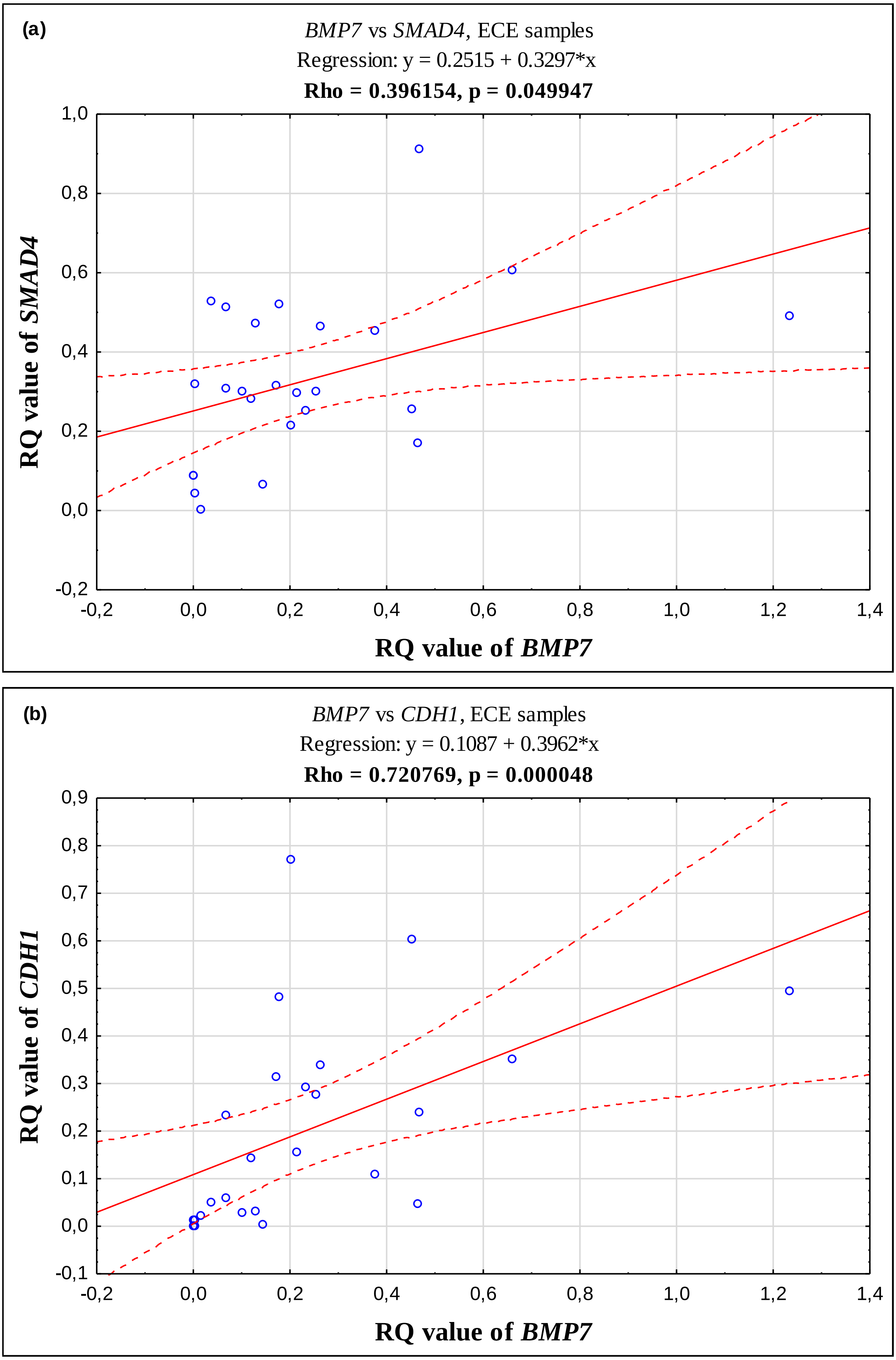
(a)
BMP7 vs SMAD4, ECE samples
Regression: y = 0.2515 + 0.3297*x
Rho = 0.396154, p = 0.049947
RQ value of SMAD4
RQ value of BMP7
1,0
0,8
0,6
0,4
0,2
0,0
-0,2
-0,2
0,0
0,2
0,4
0,6
0,8
1,0
1,2
1,4
(b)
BMP7 vs CDH1, ECE samples
Regression: y = 0.1087 + 0.3962*x
Rho = 0.720769, p = 0.000048
RQ value of CDH1
RQ value of BMP7
0,9
0,8
0,7
0,6
0,5
0,4
0,3
0,2
0,1
0,0
-0,1
-0,2
0,0
0,2
0,4
0,6
0,8
1,0
1,2
1,4

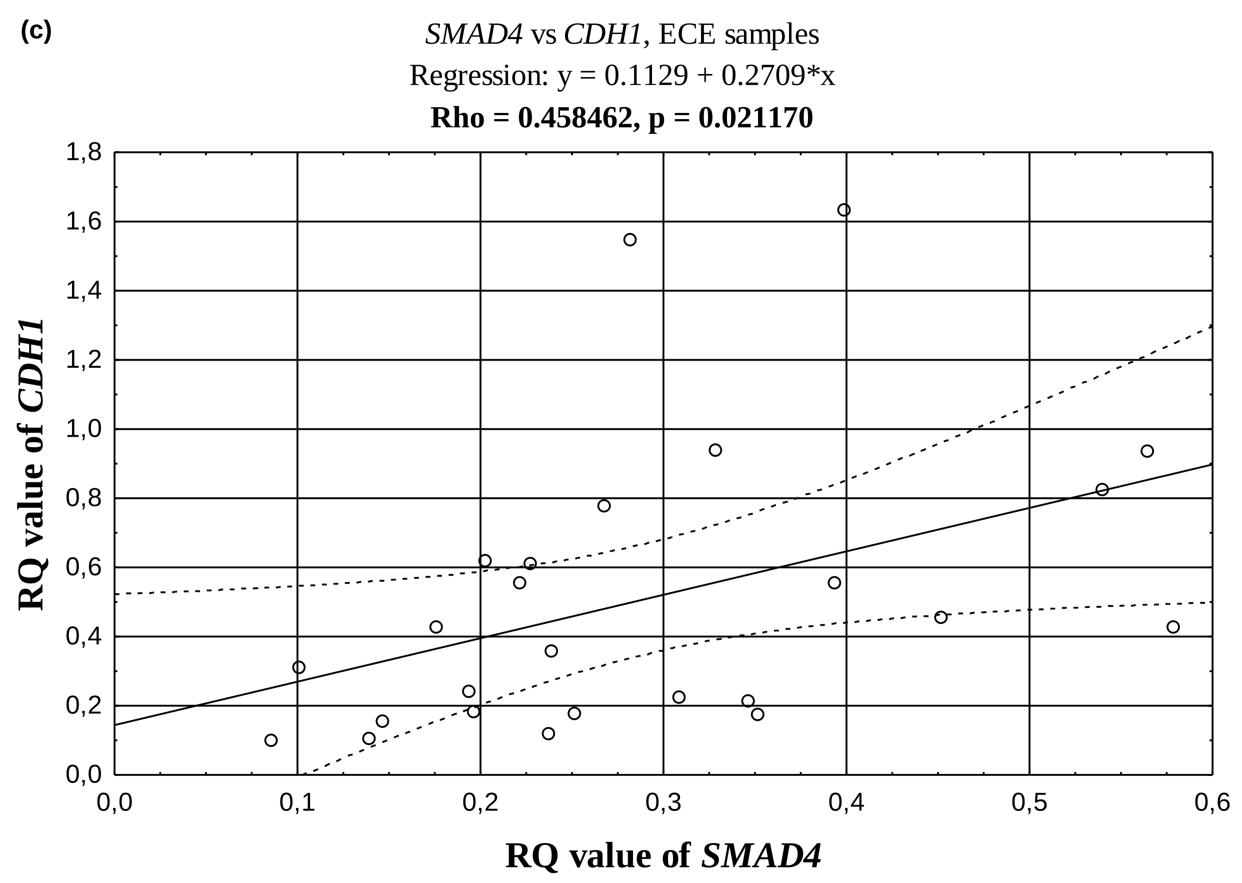
(c)
SMAD4 vs CDH1, ECE samples
Regression: y = 0.1129 + 0.2709*x
Rho = 0.458462, p = 0.021170
RQ value of CDH1
RQ value of SMAD4
0,0
0,2
0,4
0,6
0,8
1,0
1,2
1,4
1,6
1,8
0,1
0,2
0,3
0,4
0,5
0,6

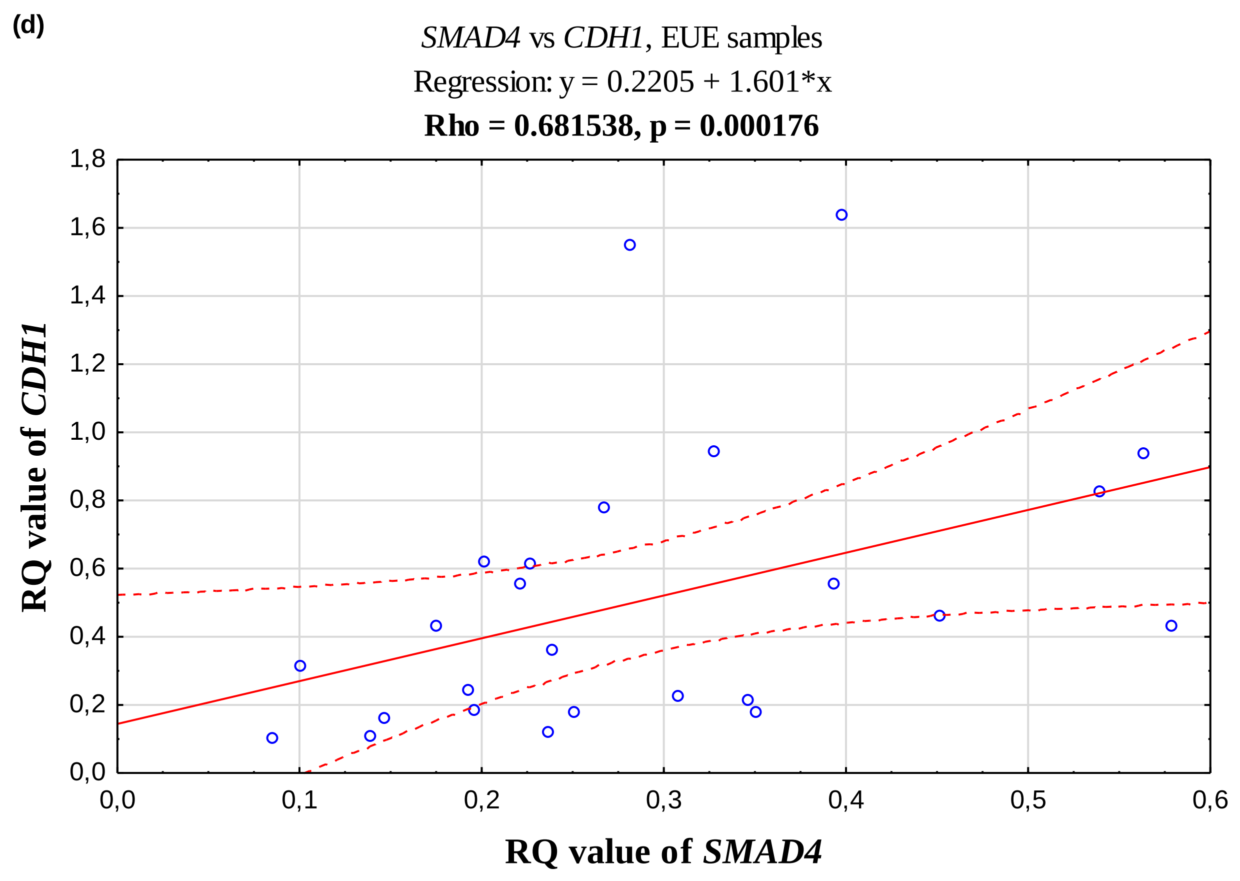
(d)
SMAD4 vs CDH1, EUE samples
Regression: y = 0.2205 + 1.601*x
Rho = 0.681538, p = 0.000176
RQ value of CDH1
RQ value of SMAD4
0,0
0,2
0,4
0,6
0,8
1,0
1,2
1,4
1,6
1,8
0,1
0,2
0,3
0,4
0,5
0,6

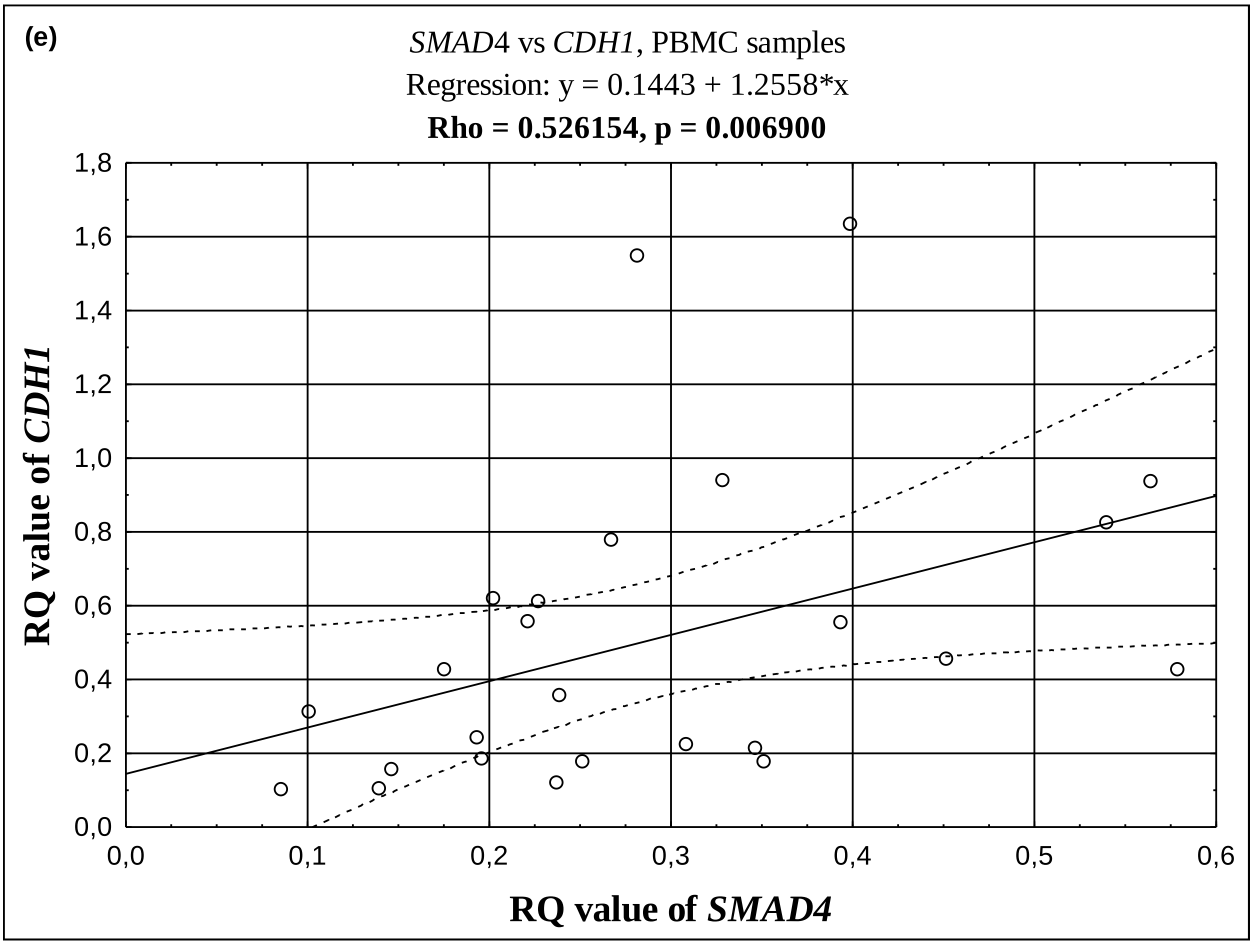

Supplementary Figure S1. The scatter plots showing correlations between expression levels (RQ values) of genes: (a), (b), (c) in ECE, (d) in EUE and (e) in PBMC samples. $p < 0.05$; Spearman's rank correlation.
